# Voice Quality Modelling for Expressive Speech Synthesis

**DOI:** 10.1155/2014/627189

**Published:** 2014-01-22

**Authors:** Carlos Monzo, Ignasi Iriondo, Joan Claudi Socoró

**Affiliations:** ^1^Computer Science, Multimedia and Telecommunication Studies, Universitat Oberta de Catalunya (UOC), Rambla del Poblenou 156, 08018 Barcelona, Spain; ^2^Grup de Recerca en Tecnologies Mèdia (GTM), Universitat Ramon Llull, La Salle, Quatre Camins 2, 08022 Barcelona, Spain

## Abstract

This paper presents the perceptual experiments that were carried out in order to validate the methodology of transforming expressive speech styles using voice quality (VoQ) parameters modelling, along with the well-known prosody (*F*_0_, duration, and energy), from a neutral style into a number of expressive ones. The main goal was to validate the usefulness of VoQ in the enhancement of expressive synthetic speech in terms of speech quality and style identification. A harmonic plus noise model (HNM) was used to modify VoQ and prosodic parameters that were extracted from an expressive speech corpus. Perception test results indicated the improvement of obtained expressive speech styles using VoQ modelling along with prosodic characteristics.

## 1. Introduction

The research fields of automatic speech recognition (ASR) and text-to-speech (TTS) synthesis benefit from expressive speech, that is, speech with emotional content being this more spontaneous, to make human-machine interactions more natural, for example, in terms of emotion recognition [1, 2] and voice transformation [3–5]. Voice quality (henceforth VoQ) and prosody parameters (*F*_0_, duration, and energy) can be conveniently manipulated to represent or convey the emotional content of speech in ASR or TTS applications respectively [1, 3, 6–10]. In spite of the fact that VoQ has been less explored than prosody, recent works propose using both types of data to improve the acoustic modelling of expressive speech [7–10]. Other studies relate perceived speech features in emotional speech to VoQ parameters [11] and deal with the association of phonation type (e.g., whispery voice) and affective speaking [12, 13].

In recent years, increasing interest has been focused on the harmonic plus noise model (HNM) [14, 15] for speech transformation [5] because high quality and versatility can be achieved. The parameterisation of speech in both harmonic and stochastic components allows for flexible manipulation of VoQ over time and pitch scales, making it possible to maintain a high degree of natural speech quality.

With the improvement in the emotional content representation of speech and the availability of improved accuracy techniques for its analysis and synthesis, interest in expressive speech synthesis (ESS) has grown [3, 6, 10, 16–21]. Along with the generation of expressive speech, it is necessary to evaluate the different existing methodologies; there is no consensus about which is the better one [22]: perceptual assessment tests with forced choices, perceptual assessment tests with free responses, or perceptual impact tests.

This paper presents the perceptual assessment carried out to evaluate the proposed expressive speech styles transformation methodology, based on prosody and VoQ modelling using an HNM for the speech analysis and synthesis. Prosody and VoQ modelling was conducted from a Spanish expressive speech corpus with five expressive styles: neutral (NEU), happy (HAP), sensual (SEN), aggressive (AGG), and sad (SAD). This perceptual assessment was performed by means of two evaluations. The first one was used for quality evaluation, and the second one was used for the assessment of the expressive styles identification. A forced-choice test with five possible answers was performed to evaluate both the perceived quality and the identification of expressive style of the utterances.

The rest of this paper is organised as follows: Section 2 shows the speech material that was used, an expressive speech Spanish corpus, and describes the corpus design, its labelling, and its subjective evaluation.  Section 3 deals with the expressive speech style transformation methodology, the HNM description, and the prosody and VoQ modelling.  Section 4 presents the perceptual assessment for the proposed transformation methodology and a discussion of the results. Finally, Section 5 contains the conclusions and an outline of future work.

## 2. Speech Material

The speech material used during the expressive speech style transformation experiments was an expressive speech corpus devoted to ESS in Spanish, developed with a twofold purpose: first, to be used for the acoustic modelling of emotional speech (prosody and VoQ) and, second, to be the speech unit database for our speech synthesiser.

### 2.1. Corpus Design

For the corpus design, we sought the help of experts in audiovisual communication from the Laboratory of Instrumental Analysis of the Autonomous University of Barcelona (LAICOM-UAB). Due to the LAICOM-UAB experience in advertising, a large textual database of advertisements, extracted from newspapers and magazines, was available. Moreover, this database was organized in different thematic categories: new technologies, education, cosmetics, automobile industry, and travels. According to the LAICOM-UAB experts, speech styles can be more easily defined according to the sentences' features for each one of these five categories [23], allowing the creation of an expressive oral corpus with good coverage of simulated expressive speech styles.

The texts for each expressive style were read by a professional female speaker in different recording sessions (stimulated speech). It was assumed that stimulated speech methodology, validated by [24], diminished the possibility of modelling informal spontaneous speech utterances while guaranteeing control of the recording conditions, the style definition, and the text design.

It is important to mention that the speaker had previously received training in the vocal patterns of each style. The phonetic features (segmental and suprasegmental) for these vocal patterns were defined by the experts of LAICOM-UAB. The use of texts from an advertising category aimed to help the speaker to maintain the desired style through the whole recording session. Therefore, the intended style was not performed according to the speaker's criteria for each sentence, but all the utterances of the same style were consecutively recorded in the same session following the previously learned pattern. Thus, the speaker was able to keep the required expressiveness even with texts whose semantic content was not coherent with the style. Moreover, LAICOM-UAB expert supervision was required through the recording in order to avoid possible deviations from the predefined style.

Five subject categories, selected from the advertising corpus, were assigned to the expressive speech styles in the following manner:

- *new technologies*: a neutral style (NEU) that transmits a certain maturity,
- *education*: a happy style (HAP) that generates a feeling of extroversion,
- *cosmetics*: a sensual style (SEN) based on a sweet voice,
- *automobiles*: an aggressive style (AGG) that transmits hardness,
- *travel*: a sad style (SAD) that seeks to express melancholy.

A set of phrases for each category was selected by means of a greedy algorithm [25], which made it possible to select phonetically balanced sentences from each subcorpus. To optimise the selection process, the required phonemes were sorted according to the occurrence rate presented by [26], which allowed the greedy algorithm to start by selecting sentences that contained less probable phonemes. Moreover, the selection of sentences similar to those previously selected was penalised by the greedy algorithm. Finally, the size of the corpus for each recorded expressive speech style is shown in Table 1.

### 2.2. Speech Labelling

The speech was labelled using segmentation and pitch marking. Segmentation is the identification of the temporal boundaries for each phoneme, and the pitch marks identify each period in the voice parts of the speech. Segmentation is related to segmental duration, whereas pitch marking is related to pitch or fundamental frequency (*F*_0_).

The alignment of the different phonemes, or segmentation, was carried out by means of forced alignment using the HTK (http://htk.eng.cam.ac.uk/) tool and the available phonetic transcription. The resulting segmentation is used in the extraction of acoustic segmental features related to both prosody and VoQ and used for recognition and synthesis purposes.

The pitch marking was based on the Robust algorithm for pitch tracking (RAPT) of [27] and the application of the pitch marks filtering algorithm (PMFA) developed by [28], which improved the robustness of the final pitch marks.

### 2.3. Corpus Subjective Evaluation

A forced answer test was designed with the question “*What emotion do you recognize from the voice of the speaker in this utterance?*” Thus, the expressive speech corpus was evaluated using a subjective test, presenting a subset of 240 utterances to 25 listeners, that produced the confusion matrix [29] presented in Table 2. The possible answers were the five styles of the corpus (see Section 2.1) plus the additional option of do not know/another (Dk/A) to avoid biasing the results in the case of confusion or doubts between two options. The risk of adding this option is that some evaluators may use it excessively to accelerate the test [30]. However, this effect was negligible in this test [9].

As a general result, the subjective test shows that all the expressive styles achieve a high percentage of identification (87.1% on average). SAD was the most highly rated (98.8%), followed by SEN (86.8%) and NEU (86.4%) styles, and finally AGG (82.7%) and HAP (81%). The confusion matrix shown in Table 2 reflects the misclassifications. It reveals that the main errors are produced in AGG (14.2% identified as HAP) and HAP (15.6% identified as AGG). Moreover, NEU is slightly confused with all the options and there is a certain level of confusion of SEN with SAD (5.7%) and NEU (4.7%). The Dk/A option was hardly used, although it was more present in NEU and SEN than in the rest of the styles. Detailed information about the subjective evaluation is presented by [9].

## 3. Expressive Speech Style Transformation

This section presents the expressive speech style transformation proposal. First of all, the harmonic plus noise model (HNM) is shown as the main processing technique used for speech analysis and synthesis. Secondly, prosody and VoQ speech parameters involved during transformations are described. Finally, the transformation methodology using HNM together with prosody and VoQ parameterisation is presented.

### 3.1. Harmonic Plus Noise Model (HNM)

Harmonic plus noise model (HNM) allows modification of the speech prosody to generate a high quality signal. In this work, we try to exploit the full capabilities of the HNM not only for prosody changes but also introducing modifications in the spectral content through VoQ parameters.

In HNM-based speech parameterisation, the voice signal *x*(*n*) can be expressed as the addition of a deterministic or harmonic component *s*(*n*) and a stochastic or noise component *r*(*n*) [14] (see (1)). The implementation used in this paper was the pitch synchronous development carried out by [31]:

(1) $$\begin{array}{c} x\left(n\right)=s\left(n\right)+r\left(n\right). \end{array}$$

The lower spectral band ([0,5000] Hz) was mainly modelled as the addition of harmonically related sinusoids (*s*(*n*)) that characterised the voiced part of the speech. For each signal frame, the deterministic part is represented by the amplitudes, frequencies, and phases of the corresponding harmonics and the analysis time instants. The number of harmonics depends on the pitch (or *F*_0_) value at each frame. The harmonic part was synthesised through overlap-add technique using triangular windows.

Unvoiced sounds and nonperiodic speech events were modelled by the stochastic component (*r*(*n*)). This was carried out by an autoregressive (AR) model in which both the spectral and temporal fluctuations were represented by constant-frame-rate, time-varying *Q*-order linear predictive coding (LPC) coefficients and variances.

With regard to the HNM analysis process, the harmonic component estimation was based on [32] algorithm for spectral peak extraction in the frequency domain, incorporating a harmonicity constraint into the frequency-based cost function by using the Lagrange multipliers optimisation method to guarantee the harmonicity of the estimated frequencies [31].

Finally, in order to perform the expressive speech style transformation process during the experiments, the prosody and VoQ models were applied using the deterministic and stochastic components. The modification was carried out by manipulating frequencies, amplitudes, phases, and noise component power according to the target prosody and VoQ model requirements.

### 3.2. Prosody Parameters

The prosody parameters involved during the transformation experiments were pitch or fundamental frequency (*F*_0_), unit duration, and unit energy. These were extracted from the expressive speech corpus presented in Section 2 and modelled using a case-based reasoning (CBR) system.

CBR is a useful data mining technique in the context of ESS [21] that returns the case that best fits the target requirements. In this way, expressive speech transformation can be enhanced using a CBR system matched to a specific expressive corpus. CBR is based on the creation of a database with different situations or cases which appeared in the corpus (memory of cases). First of all, it is necessary to identify the attributes (or features) that define the cases for the prediction of phone duration, phone energy, and the intonation contour. Then, the training set is generated by joining the prosodic parameters annotated in the speech corpus with the prosodic features extracted from the linguistic analysis of the text. A reduction of possible cases is achieved through clustering of the classes that are represented by the same attributes. The final aim of CBR is to map a solution from previous cases to the target problem. Thus, the most similar case is recovered from the database using the Minkowski metric to the selected attributes. Therefore, given the input text, the predicted parameters are fundamental frequency contour (*F*_0_), energy contour, and segmental duration.

The automatic extraction of prosodic features from text was achieved by means of the linguistic analysis tool proposed by [21] that carries out the phonetic transcription of the text using SAMPA phonetic alphabet [33], annotating intonation groups (IG), stress groups (SG), words, and syllables. Regarding IG and SG, an IG in Spanish is defined as a structure of coherent intonation that does not include any major prosodic break, and an SG is defined as a stressed word preceded, if appearing, by one or more unstressed words. With regard to prosodic breaks, they take place due to pauses or significant inflections of the *F*_0_ contour. In terms of segmental duration and energy modelling, the phone was chosen as the basic acoustic unit, and its duration depends basically on its identity and the context where it is placed, just as occurs for energy in a similar way. Finally, SG was chosen for the *F*_0_ contour modelling, incorporating the influence of the syllable and the pitch structure at IG level by means of the concatenation of SG contours.

### 3.3. Voice Quality Parameters

The VoQ parameters involved in the transformation experiments were already used and analysed in previous studies in which their usefulness for expressive speech discrimination was demonstrated [9, 10, 13, 34]. Thus, the following subset of VoQ parameters was considered for the expressive speech styles transformation experiment presented in this work.

- *Jitter* and *shimmer* describe the cycle-to-cycle variations of the fundamental period (inverse of the first harmonic's frequency) and the waveform amplitude, describing frequency and amplitude modulation noise, respectively. These parameter definitions were modified from the methodology used in tools like Praat [35] or the multidimensional voice program (MDVP) (http://www.kayelemetrics.com/index.php?option=com_product&Itemid=3&controller=product&task=learn_more&cid=56) in order to cancel prosodic interference in their measurements [34].
- *Harmonic-to-noise ratio* (HNR) describes the energy ratio between the HNM harmonic and stochastic components. The harmonic part energy is computed by the sum of the squared amplitudes of all the harmonics, while, for the stochastic part, the energy depends directly on the noise variance.
- *Hammarberg index* (HammI) is defined as the ratio between the maximum energy in the 0–2000-Hz and the 2000–5000-Hz frequency bands. Then, this parameter is computed with the maximum squared amplitude of the harmonics on each respective band of the HNM harmonic component.
- *Relative amount of energy* in the high- (above 1000 Hz) versus the low-frequency range of the voiced spectrum (pe1000): this parameter is computed with the squared amplitudes of the harmonics within high and low frequency bands of the HNM harmonic component.

### 3.4. Transformation Methodology

This section describes the proposed expressive speech transformation methodology. It is based on the previous work carried out by [10], where an initial strategy for the modification of VoQ together with prosody was proposed, using HNM for speech analysis and synthesis, to improve the perception of the transformed speech. Despite the benefits of using VoQ in combination with prosody reported in that work, a deeper analysis of the transformation methodology was considered necessary from two points of view: (i) to evaluate the identification rate improvement of the resulting transformed expressive speech and (ii) to analyse the resulting speech quality.

Initial experiments were conducted using Pitch synchronous overlap and add- (PSOLA-) based TTS to perform the VoQ modifications [34]. This algorithm is simple and straightforward when pitch, energy, and duration are modified, but some problems arise when spectral-based VoQ parameters need to be modified. Therefore, for the new experiments, the HNM was chosen as the tool for modifying and synthesising speech signals because of its flexibility, allowing such spectral modifications.

**Figure 1:**
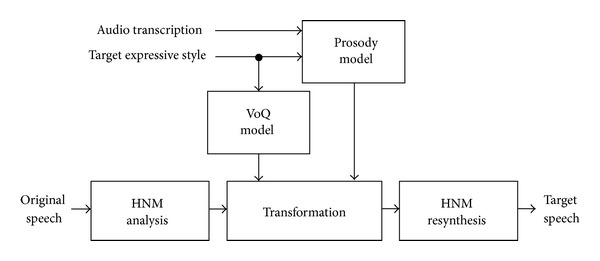
A block diagram for the proposed expressive speech styles transformation methodology.

As shown in Figure 1, the proposed block diagram for the expressive speech styles transformation methodology is divided into three main parts. First, HNM analysis and resynthesis blocks extract the original speech information and regenerate it when the HNM parameter transformation is performed. Second, prosody and VoQ are predicted through the use of a prosody and VoQ models, respectively. Prosody is predicted by means of CBR by obtaining the target information for each phoneme: *F*_0_ contour, energy contour, and segmental duration. The VoQ is modelled by using transformation rules, extracted from the analysis of VoQ parameters in the expressive speech corpus presented in Section 2, by means of mean (*μ*) and standard deviation (*σ*) parameters manipulation. Finally, the speech transformation is carried out, based on the results of the HNM analysis and the prosody and VoQ models.

Several considerations must be made to determine which parameters and their values should be involved in the transformations. For the prosody modifications, all of the parameters were involved in all of the transformations. However, the selection of the VoQ parameters and the corresponding values to be used during the transformation was based on the work of [2, 10, 13, 34] in which the following aspects were considered:

1. the results of previous studies about the use of VoQ parameters in the discrimination of expressive speech styles,
2. an exhaustive classification experiment to obtain different configurations for all parameters and expressive styles,
3. descriptive statistics calculated for all expressive styles of the corpus and all involved VoQ parameters.

Prosody modifications use the information about the original utterance and about the target from CBR predictions (see Section 3.2), so a multiplicative transformation factors among original and target parameters values were calculated and the modifications were performed. The modification of segmental durations and *F*_0_ was carried out on the HNM parameters according to the work of [31]. Nevertheless, energy modification was performed directly on the utterance audio samples by multiplying each sample by the corresponding multiplicative transformation factor [21].

With regard to VoQ parameter modification, both means and standard deviations of parameters values, predicted from the corpora presented in Section 2, were modified according to (2), obtaining the target VoQ value (VoQ_*t*_). For a given VoQ parameter, the mean (*μ*) modification was carried out by means of the original mean (*μ*_*o*_) subtraction (corresponding with the mean value for the expressive speech style) from the original parameter value (VoQ_*o*_) and, finally, the target mean (*μ*_*t*_) for this parameter was added (corresponding with the mean of the target expressive speech style). Regarding the standard deviation (*σ*), a multiplicative transformation factor per VoQ parameter and target expressive style, calculated as the ratio between the target (*σ*_*t*_) and the original standard deviation (*σ*_*o*_), was used in order to vary the intensity of the current parameter. In order to obtain more robust measurements, following the proposals of [3, 36], only vowels were considered in these computations. This proposal for the transformation methodology will let us evaluate the usefulness of combining VoQ together with prosody with the aim of improving the obtained expressive speech style identification rate maintaining an acceptable speech quality:

(2) $$\begin{array}{c} \text{Vo}{\text{Q}}_{t}=\frac{\sigma_{t}}{\sigma_{o}}\cdot \left(\text{Vo}{\text{Q}}_{o}-\mu_{o}\right)+\mu_{t}. \end{array}$$

The target VoQ values were obtained applying the presented transformation to the original VoQ parameters values frame-by-frame. This VoQ parameter modification using the HNM, performed according to the work of [37], is described below.

- *Jitter*: only the frequencies for the HNM harmonic component are modified. Once the *F*_0_ curve is obtained from the CBR prosody prediction module, slow *F*_0_ variations are removed to avoid interference due to prosodic information, and the new *F*_0_ microprosody variations related to jitter are applied. New jitter variance is obtained by means of the presented transformation methodology, and the final pitch curve is computed adding the new jitter to the previously extracted slow *F*_0_ variations [34].
- *Shimmer*: the modification of this parameter is directly applied to the time-domain waveform. The same process used for jitter modification has been applied to modify the shimmer. However, pitch synchronous peak-to-peak amplitude variations curve is used instead of *F*_0_ contour information [34].
- *HNR*: multiplicative transformation factors, calculated as the ratio between target and original HNR values, are applied in the HNM harmonic and stochastic components to guarantee the desired energy ratio and the total energy after the transformation. For each signal frame, the multiplicative transformation factor in the harmonic part is the same for all harmonic amplitudes, and, in the stochastic part, it affects the noise variance. An additional energy correction factor for both components is finally applied to maintain the original frame energy in the transformed signal.
- *HammI*: only the maximum harmonic amplitude of each frequency band (the 0–2000-Hz and the 2000–5000-Hz frequency bands) in the HNM harmonic component is modified according to the target parameter value (using a transformation factor measured as the quotient between the target and original HammI values). An additional energy correction factor, the same for each frequency band, maintains the original frame energy during the transformation. The HNM stochastic component is not manipulated.
- *pe1000*: using the corresponding multiplicative transformation factor calculated as the relation between target and original pe1000 values, the ratio between the HNM harmonic component energy of the [0,1000] Hz and [1000,5000] Hz frequency bands is modified. A multiplicative constant factor, specific for each band, is applied to the harmonic amplitude values without any manipulation of the HNM stochastic component. The same global energy normalization procedure used in previous parameters is finally carried out.

Due to the tight relation among VoQ parameters, a change in a parameter during the overall modification can affect another one, especially when they model similar low-level signal features (i.e., spectral bands). For example, a change in HammI can produce a variation in pe1000 parameter as they measure energies in the same spectral bands. In order to minimize the impact among them, and taking into account the modification needs for each one, the following order modification was proposed by [37]: (1) Jitter, (2) HNR, (3) pe1000, (4) HammI, and (5) Shimmer.

Table 3 presents the VoQ parameters involved in each transformation from neutral to the target expressive style. Nevertheless, as was said previously, all parameters were not involved during all the transformations, since the best parameters identifying to each expressive speech style were found out from previous conducted work in discriminative analysis, expressive speech styles classification using VoQ, and descriptive statistics of corpus. Moreover, Table 4 shows mean (*μ*) and standard deviation (*σ*) values for all VoQ parameters extracted from the expressive speech corpus, used during the VoQ transformation to calculate the target VoQ parameters.

Different conclusions can be extracted from Tables 3 and 4. First, for all transformations, the HammI and pe1000 parameters were used, controlling the tension effect in the voice, showing a phonation effort or relaxation. For example, HAP and AGG styles present values for these parameters that show high energy in high frequency band, thus producing a higher perceived vocal effort in the final speech. Nevertheless, for SEN and SAD styles, the presented speech is more relaxed. Second, jitter and shimmer parameters let us control the quivering voice effect, and so their use is more remarkable in SEN and SAD styles. Finally, the control of the amount of noise which appeared in the speech is carried out by means of HNR parameter, useful during SEN style identification.

To sum up, this prosody and VoQ transformation methodology entails modifying the HNM parameters, that is, frequencies, amplitudes, and phases for the harmonic component and variance for the stochastic component. Therefore, two kinds of transformations were carried out. The first one, the prosody modification, was guided through the CBR system so that it affected only the prosody; thus, the frequencies, amplitudes, and phases of the HNM were modified to produce the required *F*_0_ contour, energy contour, and segmental duration. The second one, the VoQ parameter modification, also modified the HNM parameters, but in the specific way previously described and briefly summarized below.

The jitter parameter also controls the *F*_0_ contour; this way, the frequencies, amplitudes, and phases were also affected because vocal tract observations are highly related to the pitch frequency. In the shimmer parameter case, all the modifications were carried out directly on the time-domain speech signal [34]. The HammI and pe1000 parameters are related to ratios or approximations of energy in different frequency bands of the harmonic component. Therefore, the HNM amplitude vector was modified. To vary the HNR parameter, both HNM harmonic and stochastic components were modified by manipulating the harmonic amplitudes and stochastic variances, respectively. For HammI, pe1000, and HNR parameters, the variation multiplicative transformation factor was distributed between two spectral bands (in the HNM harmonic component for HammI and pe1000) or both components (the HNM harmonic and stochastic components for HNR), ensuring that speech energy was preserved when the transformation was performed.

## 4. Perceptual Assessment

In the work of [10], the utility of using a combination of prosody and VoQ for ESS was demonstrated by means of a comparison mean opinion score (CMOS) [38] perceptual test. However, the quality of the final transformed expressive speech and, especially, the identification rate for each of these styles were not studied. In this section, how the parameter modification affects the the quality of the generated speech and the identification rate obtained for each target style are analysed. Thus, the transformations and the proposed methodology are validated.

### 4.1. Experiment Description

With the aim of analysing the effectiveness of the proposed transformation, a comparison between modifying prosody and VoQ parameters using the HNM technique was conducted in the following experiments, in which the quality of the generated speech and the target expressive styles identification rate were evaluated (see Sections 4.2 and 4.3, resp.). The results were compared by taking into account different configurations of parameters to be transformed: modifying only prosody, prosody plus jitter and shimmer, and, finally, prosody plus the combination of VoQ parameters for each expressive style. Each of the configurations under testing is described next, indicating the name that identified them during the evaluations.

- *“Natural”*: natural speech. A set of utterances was directly extracted from the corpus for each expressive style.
- *“ResHNM”*: HNM-based direct resynthesis of the natural utterances for each expressive style. The process of analysis and synthesis was carried out from corpus examples without applying any modification to the speech parameters.
- *“HNMPro”*: prosody transformation based on the HNM. The utterances, originally expressed in a neutral expressive style, were transformed using the prosody models.
- *“HNMProJiSh”*: transformation of prosody, jitter, and shimmer, based on the HNM. These parameters were transformed from the prosody models and by using the transformation values for jitter and shimmer, learned from the expressive corpus to transform the utterances originally expressed in a neutral expressive style. This configuration is interesting because this transformation can be conducted by means of both HNM-based and other synthesis algorithms-based TTS (e.g., PSOLA).
- *“HNMProVoQ”*: prosody and full VoQ modification based on the HNM. The utterances, originally expressed in a neutral expressive style, were transformed using the prosody models and the VoQ parameter configurations (see Section 3.4) into the target expressive style under testing.

First, the speech quality was evaluated using utterances transformed from a neutral subcorpus into happy (HAP), sensual (SEN), aggressive (AGG), and sad (SAD) expressive styles. The evaluation was carried out by means of a mean opinion square (MOS) test [38] with five possible answers, with a score between 1 and 5, in which 5 was the maximum quality and 1 was the minimum: “Excellent” (5), “Good” (4), “Fair” (3), “Poor” (2), and “Bad” (1). The results analysis was performed by grouping the five kinds of configurations into two sets:

1. natural speech (“Natural”) and resynthesised speech (“ResHNM”), which had the maximum reference values because they were real cases (natural) and also represented the best possible results that a TTS system could obtain using the HNM algorithm (resynthesis).
2. The proposed transformation methodology with the configuration of interest (“HNMProVoQ”) was compared with the rest of the configurations. The different configurations for the transformation were (i) only prosody modification (“HNMPro”), (ii) a combination of prosody and only jitter and shimmer VoQ parameters (“HNMProJiSh”), and (iii) a combination of prosody and the selected VoQ parameter for each transformation (“HNMProVoQ”). Hence, the quality changes of the HNM algorithm were evaluated.

Second, we performed the target expressive speech style identification assessment, destined to validate the proposed expressive speech transformation methodology. This was carried out through a test with 5 possible answers, with 4 of them corresponding to the target expressive styles (happy, sensual, aggressive, and sad) and a fifth category corresponding to “Others.” This fifth category was created without being assigned to any particular style to avoid an opinion bias towards the rest of the options when the answer was not clear or when no evaluated style was perceived. In an analogous way, the configuration of interest “HNMProVoQ” was also compared with the rest of the possible options in order to point out the possible differences in the modified speech parameters (prosody and VoQ).

The comparison of prosody and VoQ parameter modification strategies is interesting from the point of view that the variations of quality for a certain identification rate of the expressive speech styles can be known, and two main situations can be considered. On one hand, in the case of achieving high quality and a low identification rate, a situation can be determined in which it is necessary to improve the expressive styles models (this could be performed using more or different parameters). On the other hand, if the quality is low and a high identification rate is obtained, the speech generation algorithm will be taken into account by analysing whether it can support the desired parameter transformation and whether this transformation must be so demanding that it causes quality degradation (in this case, we could work on modifying the transformation needs).

The quality evaluation and the expressive speech styles identification test were carried out by answering two questions for the same presented utterance: “*Assess the global audio quality*” for the speech quality evaluation and “*Indicate which expressive style is transmitted by the audio*” to identify the transmitted expressive style. For these evaluations, 100 utterances were generated, containing the same number of examples for every configuration and expressive style. The total number of listeners was 17, with ages ranged between 24 and 50 years old. There were 13 males and 4 females. Out of the group, 8 of them were experts on speech technologies.

With regard to the test utterances, they were selected according to the type of the conducted test and the applied configuration. For “Natural” and “ResHNM,” five utterances for each subcorpus were selected. Regarding their characteristics, a variety of intonation patterns were used by selecting declarative, interrogative, and exclamation expressions, with a mean duration of 4.5 seconds for NEU, 4 seconds for HAP, 3.4 seconds for SEN, 3 seconds for AGG, and 4.5 seconds for SAD. For the rest of tests, the same NEU utterances were transformed into the different expressive speech styles. Detailed information about the selected sentences is presented by [37].

Next, the obtained results and conclusions are presented for every configuration and expressive style. The results for the quality assessment are shown first (Section 4.2), and the subjective identification results are presented second (Section 4.3).

### 4.2. Subjective Quality Assessment

**Figure 2:**
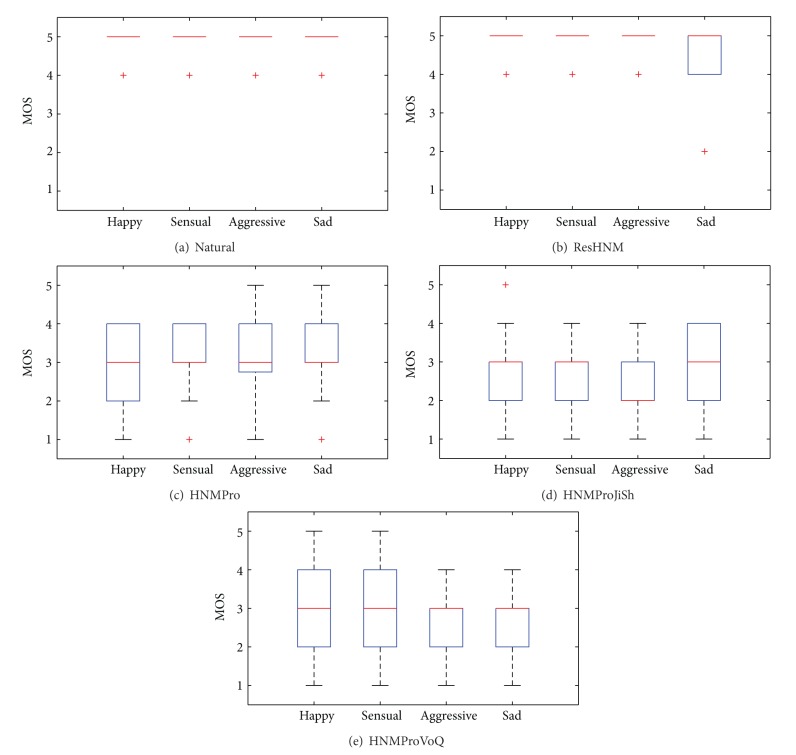
Quality MOS test results for the configurations of “Natural,” “ResHNM,” “HNMPro,” “HNMProJiSh,” and “HNMProVoQ.”

The boxplots [39] of Figures 2(a) and 2(b) show the quality assessment results for reference configurations: “Natural” (see Figure 2(a)) and “ResHNM” (see Figure 2(b)). The high quality of these utterances “Excellent” can be observed; only the sad style, with HNM-based resynthesis, presented certain result dispersion. These results are consistent with the configurations that were used; due to this, in general terms, HNM-based synthesis highly depends on the resulting speech parameterisation, so it is more affected by pitch marks than other TTS-based algorithms like PSOLA, as well as the HNM parameter estimation of the deterministic and stochastic components. For example, in the case of the sad style (see Figure 2(b)), its specific acoustic characteristics (e.g., tremulous voice) could lead to more analysis inaccuracies, causing more synthesis artefacts.

Once the reference configurations were analysed (“Natural” and “HNMPro”), the results for the evaluated transformation-based configuration were studied (see Figures 2(c)–2(e)) with the final goal of going into a deeper analysis of the transformation methodology of interest (“HNMProVoQ”).

First, the quality values for “HNMPro” (see Figure 2(c)) is presented. A certain quality degradation due to the application of expressive prosody on the neutral utterances could be observed. Notice that the quality value is centred on “Fair.”

The resulting MOS values for the evaluation of “HNMProJiSh” configuration are analysed in Figure 2(d). The quality value for the HNM was maintained practically constant at the median (“Fair”), except for the aggressive case (“Poor”). Then, the HNM clearly becomes a good option in the transformation of expressive speech styles because it offers an acceptable final quality despite the parameters modification.

Finally, we evaluated the quality obtained by the “HNMProVoQ” configuration (see Figure 2(e)) in which the VoQ transformations were matched to the necessities of each target expressive speech style (see Table 3). Figure 2(e) shows how the quality is maintained between acceptable values (“Fair”), as occurred in the configuration of the HNM in which only the prosody transformation was involved (see Figure 2(c)). This fact shows that the HNM makes it possible to introduce VoQ transformations without decreasing the quality. It is also notable that the quality for the happy, sensual, and aggressive expressive styles increased with regard to the transformation of prosody together with only jitter and shimmer (see Figure 2(d)). There was a slight decrease in the sad style, which had already occurred in the reference case (see Figure 2(b)).

With these results, we can conclude that the VoQ transformations could be used to improve the identification rate of expressive styles (see Section 4.3) during the speech synthesis, maintaining the speech quality regarding the well-known prosody modelling. Nevertheless, excess signal manipulation can bring about negative effects too, decreasing the quality. For example, in the case of the sad expressive style, as it is shown in Section 4.3, the best interclass identification rate was achieved (see Table 5), although it was not the style with the best perceived quality (see Figure 2(e)).

### 4.3. Subjective Expressive Speech Style Identification

Once the quality assessment results have been analysed and discussed, we will analyse the results that were obtained from the expressive speech style identification subjective test. In this case, the aim was to evaluate the identification degree of the synthesised style that was achieved using the proposed method, related to the main goal of this work of validating the usefulness of VoQ in the enhancement of expressive synthetic speech for style identification. We have to remember that the transformation was carried out from the neutral style into another style (happy, sensual, aggressive, and sad). In the performed test, listeners could choose any of these four expressive styles and the option “Others,” which avoided biasing the measure towards any of them.

The subjective identification results are presented by means of a confusion matrix (%) [29] and *F*1 measures [40]. First, the confusion matrix informs us about how good the identification was, and above all, it lets us detect any existing confusion among the expressive styles. Second, the *F*1 measure gives a more real and compact vision about how good the identification was, taking into account both the correct classified cases and the existing confusion among styles. This measure is the harmonic mean of precision and recall. For a studied style, the precision is defined as the number of cases correctly classified divided by the total number of cases classified in that style. The recall is defined as the number of cases correctly classified divided by the total number of existing cases that should have been classified within that class.

As was done for the quality evaluation, first, the results obtained for natural speech (“Natural”) and HNM-based resynthesis (“ResHNM”) are shown (see Table 5). It was observed that both configurations produced similar results, as was expected. Some confusion existed between happy-aggressive and sensual-sad styles. The *F*1 values were greater than 0.92 for all of the expressive styles in both configurations.

The next step was analysing how the neutral style transformation into the target expressive styles affected the identification of the desired styles (see Table 5). First, we wanted to study the limitation of each transformation and the improvement of the perception of the expressive styles from the use of VoQ parameters when using only prosody, corresponding to the first transformation attempt from the prosody parameters (“HNMPro” configuration). Second, the results for combining prosody together with VoQ are presented in the “HNMProJiSh” (prosody with jitter and shimmer) and “HNMProVoQ” (prosody and the proposed configuration for VoQ parameters) configurations. The improvement of *F*1 values using the interest configuration (“HNMProVoQ”), regarding the rest of HNM transformation configurations (“HNMPro” and “HNMProJiSh”), is summarised in Table 6.

The results obtained with the first configuration, in which the speech signals were only modified to achieve the predicted prosodic parameters using the HNM algorithm (“HNMPro”), are shown in Table 5. Notice that the identification *F*1 values have declined dramatically from their references for all styles. The sensual style case obtained the best result with regard to the rest of styles (*F*1 = 0.41).

Once the results for the prosodic transformation were reviewed, the inclusion of jitter and shimmer VoQ parameters was evaluated (“HNMProJiSh”). In this case, the results (see Table 5) show that the identification rate remained stable for the HNM (except for the sad style, which was improved to a value of *F*1 = 0.48). Although the identification levels were still low, the results were better for the sad style (*F*1 = 0.48), coinciding with the maximum quality for this configuration (see Figure 2(d) in Section 4.2), possibly because of the stability demonstrated by the HNM during the parameter transformation; each style could be characterised without adding dispersion.

The last configuration to be analysed is “HNMProVoQ,” in which both prosody parameters and selected VoQ configurations were involved during the transformation of expressive speech styles (see Table 5). The first observation to emphasise is that we obtained the best results (see Table 6) with regard to the rest of configurations involving parameter transformations (“HNMPro” and “HNMProJiSh”). The second thing to note is the good result obtained for the sad style (*F*1 = 0.56), followed by the sensual style (*F*1 = 0.48). This is particularly important if we take into account the existing confusion between both styles in the reference (“Natural” and “ResHNM”). The value obtained for the happy style (*F*1 = 0.42) is very interesting too, especially because of the progression regarding the use of only prosody and jitter and shimmer. Finally, the aggressive style yielded the worst absolute result (*F*1 = 0.39), although it yielded the highest increment in its identification regarding “HNMProJiSh” configuration (44.4% according to Table 6).

The main reason for the identification error is the existing confusion between happy-aggressive and sensual-sad styles, which already appeared in the reference values (“Natural” and “ResHNM”). There is some general confusion towards the sad and “Others” categories. Once the test was finished, the listeners could write their comments. From them, it was observed that “Others” was in general related to the detection of a neutral style (i.e., the source style). Therefore, a higher modification for the parameters during the transformation is necessary. Thanks to the stability demonstrated by using the HNM, both for the quality (see Figures 2(c), 2(d), and 2(e)) and the identification (see Table 5), the level of parameter transformation could be increased. Moreover, according to listener observations, the trend towards the transmitted style identification using the semantic content of the utterance was also detected (i.e., what is the sentence talking about?), which caused a bias towards the wrong styles, especially in those cases in which acoustic characteristics could not identify them clearly (e.g., a whispering or quivering voice in the sensual or sad styles, resp.).

As a conclusion, both from the quality and the identification perspectives, when the best identification rate was achieved through the use of prosody together with a combination of VoQ (e.g., aggressive and sad styles), the quality levels went worse (see Figure 2(e) in Section 4.2). However, when the signal manipulation was not so high, the quality was maintained more constant (e.g., happy and sensual styles), and the style identification improvement was not so significant. Therefore, an agreement could be necessary between the expected quality and the amount of VoQ parameter modification needed for the identification of the style. In order to achieve this, a more sophisticated prediction of target VoQ parameters, similar to the one conducted on prosody modelling, could be necessary.

## 5. Conclusions and Future Work

The main aim of this work was to validate the usefulness of VoQ in the enhancement of expressive synthetic speech for style identification presenting an acceptable quality. The harmonic plus noise model (HNM) of expressive style transformation based on prosody and voice quality modifications was evaluated by means of a perceptual assessment of speech quality and expressive speech styles identification. With regard to this methodology, first, flexible HNM parameterisation was used to extract the fundamental speech parameters that must be used during the performed prosody and VoQ modifications. Second, the prosody parameters were predicted by means of the CBR system and modified using the HNM parameters and the acoustic waveform, being a first attempt at the expressive speech style transformation. Finally, once prosody was transformed, the VoQ parameters were modified by varying the HNM parameters and the acoustic waveform according to the available VoQ models. To select which VoQ parameters should be modified, an analysis of the best configurations was previously performed.

The test for the perceptual assessment was carried out for different configurations, including natural speech and speech synthesis using the HNM: speech resynthesis, prosody modification, prosody plus jitter and shimmer modification, and, finally, prosody together with the best VoQ configurations using the HNM (the configuration of interest). These analyses resulted in an acceptable speech quality during the transformation. In addition, the expressive styles identification rate was directly related to the results of the quality test, which reported the best results for the configuration of interest.

To summarise, from the perceptual assessment of both the quality and identification experiments, the following conclusions can be drawn about the expressive speech styles transformation viability. First, the use of speech analysis and synthesis by means of the HNM made it possible to achieve good quality and speech manipulation control during the transformations of both prosody and VoQ parameters. Second, the combination of prosody and VoQ parameters produced significant improvements in the expressive speech styles identification rate compared with only using prosody and a subset of VoQ parameters. Finally, from the comments of listeners during the test, it was observed that the semantic content of utterances could be a limitation for the expressive speech style identification.

In spite of the good results, more work is needed. A better model for VoQ is necessary, as one for the prosody already exists, in order to improve the model for each expressive speech style contained in the corpus. Moreover, in this way, distinguish pairs of expressive styles with identification difficulties let us improve their identification rate.

Finally, the results obtained both for quality and identification encouraged us to continue with the modelling of prosody and VoQ using HNM speech analysis and synthesis.

## Figures and Tables

**Table 1:** Number of sentences and duration, per expressive speech style, for the expressive corpus.

	Number of sentences	Duration (min)
NEU	833	50
HAP	916	56
SEN	841	51
AGG	1048	84
SAD	1000	86

**Table 2:** Average confusion matrix (%) for the subjective test (the maximum correct classification value is in bold).

(%)	NEU	HAP	SEN	AGG	SAD	Dk/A
NEU	86.4	1.3	3.6	5.3	0.7	2.7
HAP	1.9	81.0	0.2	15.6	0.1	1.2
SEN	4.7	0.1	86.8	0.0	5.7	2.6
AGG	1.8	14.2	0.1	82.7	0.1	1.1
SAD	0.5	0.0	0.6	0.0	98.8	0.1

**Table 3:** Voice quality selected parameters during neutral-target transformations (“•” when the parameter is selected and “—” otherwise).

	Jitter	Shimmer	HNR	HammI	pe1000
HAP	—	—	—	•	•
SEN	•	•	•	•	•
AGG	•	•	—	•	•
SAD	•	•	—	•	•

**Table 4:** Mean (*μ*) and standard deviation (*σ*) values for all VoQ parameters extracted from the expressive speech corpus.

*μ*/*σ*	Jitter	Shimmer	HNR	HammI	pe1000
NEU	0.21/0.36	0.06/0.19	25.68/4.28	28.37/7.68	−14.35/6.41
HAP	0.24/0.30	0.03/0.06	24.06/5.26	25.12/7.81	−9.29/8.96
SEN	0.49/0.74	0.11/0.22	24.30/6.09	30.98/8.08	−16.08/7.58
AGG	0.14/0.12	0.03/0.06	23.45/4.45	23.69/7.31	−6.15/8.80
SAD	0.10/0.08	0.12/0.20	29.38/6.58	35.77/8.78	−16.97/8.80

**Table 5:** The confusion matrix (%) and *F*1 measures in the expressive speech styles identification for the reference configurations (“Natural” and “ResHNM”) and HNM transformation configurations (“HNMPro,” “HNMProJiSh,” and “HNMProVoQ”).

(%)	HAP	SEN	AGG	SAD	Others
Natural					
HAP	94.1	0.0	5.9	0.0	0.0
SEN	0.0	100.0	0.0	0.0	0.0
AGG	0.0	0.0	100.0	0.0	0.0
SAD	0.0	9.4	0.0	90.6	0.0
(*F*1)	* 0.97 *	* 0.96 *	* 0.97 *	* 0.95 *	*— *

ResHNM					
HAP	87.1	0.0	11.8	0.0	1.2
SEN	1.2	95.3	1.2	2.4	0.0
AGG	1.2	0.0	98.8	0.0	0.0
SAD	0.0	11.8	0.0	88.2	0.0
(*F*1)	* 0.92 *	* 0.92 *	* 0.93 *	* 0.93 *	* — *

HNMPro					
HAP	30.6	3.5	17.6	28.2	20.0
SEN	1.2	31.8	9.4	40.0	17.6
AGG	35.3	1.2	21.2	23.5	18.8
SAD	2.4	18.8	10.6	41.2	27.1
(*F*1)	* 0.36 *	* 0.41 *	* 0.27 *	* 0.35 *	*— *

HNMProJiSh					
HAP	30.6	3.5	16.5	27.1	22.4
SEN	3.5	30.6	4.7	36.5	24.7
AGG	32.9	2.4	18.8	22.4	23.5
SAD	4.7	17.6	1.2	58.8	17.6
(*F*1)	* 0.36 *	* 0.40 *	* 0.27 *	* 0.48 *	*— *

HNMProVoQ					
HAP	34.1	3.5	25.9	14.1	22.4
SEN	0.0	40.0	5.9	34.1	20.0
AGG	23.5	2.4	31.8	16.5	25.9
SAD	3.5	20.0	1.2	64.7	10.6
(*F*1)	* 0.42 *	* 0.48 *	* 0.39 *	* 0.56 *	* — *

**Table 6:** *F*1 measure improvement percent (%) using “HNMProVoQ” compared with “HNMPro” and “HNMProJiSh” transformation configurations.

(%)	HAP	SEN	AGG	SAD
HNMPro	16.7	17.1	44.4	60.0
HNMProJiSh	16.7	20.0	44.4	16.7
